# Measurement Grid Optimization for OTA Testing of 5G Smart Watches

**DOI:** 10.3390/s25103185

**Published:** 2025-05-19

**Authors:** Xudong An, Fei Liu, Meijun Qu, Siyang Sun

**Affiliations:** 1 School of Electronic Engineering, Beijing University of Posts and Telecommunications, Beijing 100876, China; anxudong@caict.ac.cn; 2 China Academy of Information and Communications Technology, Beijing 100191, China; 3 School of Information and Communication Engineering, Communication University of China, Beijing 100024, China; 202220080904008@mails.cuc.edu.cn (F.L.); qumeijun@cuc.edu.cn (M.Q.)

**Keywords:** 5G, smart watches, measurement grid optimization, MU analysis, OTA

## Abstract

Over-the-air (OTA) testing is crucial for optimizing wireless performance of 5G smart watches and improving their user experience. However, the current required test time is so long that it is almost impossible to complete the entire OTA testing without recharging and repositioning, which is unacceptable for the industry. Therefore, test-time reduction is significant. The objective of this work is to optimize measurement grids for OTA testing of 5G smart watches, which balance accuracy with efficiency. In this research, passive patterns from a typical 5G commercial smart watch are measured at different bands as reference patterns, which represent general radiation properties of 5G commercial smart watches. The effect of various coarse grids on OTA testing precision is characterized quantitatively by analyzing their accuracy in reconstructing reference patterns. The related measurement uncertainty (MU) terms are then evaluated and determined quantitatively based on statistical analysis. According to the derived MU limits for grid configurations, reducing grid points from currently required 62 (30/30) to 26 (45/45), and from 266 (15/15) to 62 (30/30) could save roughly 60% and 75% of the test time, respectively, with an uncertainty increase of 0.1 dB for both Total Isotropic Sensitivity (TIS) and Total Radiated Power (TRP) testing, which is considered acceptable. Furthermore, the feasibility of the proposed MU analysis and recommended grids have been experimentally verified.

## 1. Introduction

In the 5G era, smart watches play a crucial role in areas such as health management, message notification, electronic payment, navigation and positioning, and social entertainment, with their convenience, versatility, and seamless connection with smartphones. For the use scenario of smart watches, the reliability, coverage, response speed, and security of wireless connections are key factors that affect user experience and system performance, which are heavily dependent on agile and high-performance antennas [1,2]. Therefore, OTA testing, which can accurately characterize the wireless performance of devices under test (DUTs) in real-life usage environments, is of great significance for optimizing the wireless/antenna performance of smart watches and improving their quality of user experience [3,4,5,6,7,8,9]. OTA testing has been mandated as the standard method for wireless performance evaluation of DUTs by the Cellular Telecommunications and Internet Association (CTIA) and the 3rd Generation Partnership Project (3GPP), as well as network operators.

**Figure 1:**
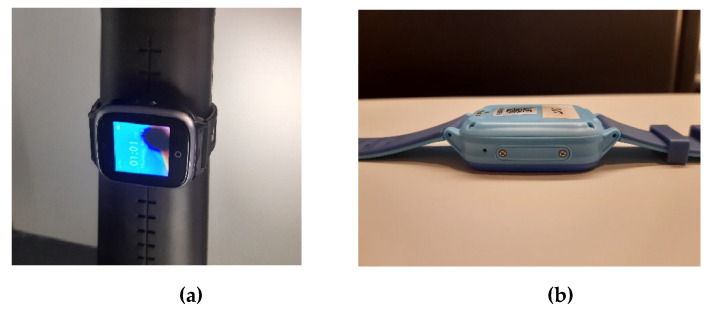
Illustration of the influence of excessive heat on smart watch: (**a**) the hot spot noticed during the OTA testing; (**b**) deformed case after OTA testing.

For 5G Sub-6 GHz user equipment (UEs), the currently required lowest measurement grid resolutions in both CTIA and 3GPP specifications are 15° and 30° for Total Radiated Power (TRP) and Total Isotropic Sensitivity (TIS) testing, respectively [10,11,12,13,14]. Generally, it takes around 60 and more than 120 min for cellular TRP and TIS testing, respectively. For 5G Assisted Global Navigation Satellite System (A-GNSS) TIS testing, this figure could be increased to more than 240 min. Moreover, during both TRP and TIS testing, DUTs are required to be powered by their own batteries and transmit at maximum output power. For smartphones and notebooks with relatively larger batteries, this testing method and the associated amount of test time seem acceptable. However, for smart watches with relatively low-capacity batteries due to limited space and working volume, it is impossible to complete the entire testing without recharging and repositioning due to battery depletion, which would lengthen the test time that is already considered too long to an unacceptable level. On the other hand, smart watches are designed to transmit at full power for only a short duration within each hour, rather than for long operation, and as a result, they typically have a very small heatsink for the power amplifier to dissipate the heat. As a practical matter, attempts to perform OTA testing per current CTIA and 3GPP specifications for mobile phones will, in many cases, cause the DUTs to thermally shut down one or more times during a TIS test, and the DUTs may even be damaged due to excessive heat. Figure 1 presents an example of the influence of excessive heat associated with the cellular transmitter during a TIS test. The hot spot can be noticed in Figure 1a during the TIS testing on the screen while the watch deformed apparently after the TIS testing. As a result, the test-time reduction for OTA testing is of great significance for 5G smart watches. Unfortunately, no relevant research has been performed or published yet.

The objective of this work is to optimize measurement grid configurations for OTA testing of 5G smart watches based on the measurement uncertainty (MU) analysis for grid point reduction. In this paper, passive patterns from a typical 5G commercial smart watch are measured at different bands using a very fine grid resolution as the reference patterns, representing general radiation properties of 5G commercial smart watches. Subsequently, a variety of coarse grid configurations are compared through simulations quantitatively to determine the extent to which they can accurately reconstruct those reference patterns in order to evaluate the errors and MU associated with the grid point reduction. The relevant MU terms are then analyzed and determined quantitatively based on statistical analysis. Ultimately, constant step grids with angular resolutions of 45/45 and 30/30 are recommended for TIS and TRP testing of 5G smart watches, achieving approximately 60% and 75% test-time reduction, respectively.

## 2. Measurement Uncertainty Analysis with Grid Point Reduction

For OTA testing, the TRP (TIS) is the weighted sum of Effective Isotropic Radiated Power, EIRP (Effective Isotropic Sensitivity, EIS^−1^) values sampled over specified points (theta/phi) across the integration sphere. For a complete sphere with N theta intervals and M phi intervals, respectively, both are measured with even angular spacing, the TRP and TIS can be numerically calculated as follows:

(1) $$TIS≅\frac{2M}{\sum_{i=0}^{N}\omega \left(\theta_{i}\right)\cdot \sum_{j=0}^{M-1}\left[\frac{1}{EIS_{\theta }\left(\theta_{i},\varphi_{j}\right)}+\frac{1}{EIS_{\varphi }\left(\theta_{i},\varphi_{j}\right)}\right]}.$$

(2) $$TRP≅\frac{1}{2M}\cdot \sum_{i=0}^{N}\sum_{j=0}^{M-1}\left[EIRP_{\theta }\left(\theta_{i},\varphi_{j}\right)+EIRP_{\varphi }\left(\theta_{i},\varphi_{j}\right)\right]\cdot \omega \left(\theta_{i}\right)$$

(3) $$\theta_{i}=i\Delta \theta \;\mathrm{where}\;\Delta \theta =\frac{\pi }{N}$$

(4) $$\varphi_{j}=j\Delta \varphi \;\mathrm{where}\;\Delta \varphi =\frac{2\pi }{M}$$

where EIS and EIRP are Effective Isotropic Sensitivity and Effective Isotropic Radiated Power values sampled at each polarization and grid point, respectively, while *w*(*θ_i_*) is the theta-dependent weighting function. In this work, the Clenshaw–Curtis quadrature in Equation (5) is utilized for TRP/TIS calculations [14]. Since TIS testing takes significantly more test time compared to that of the TRP, prioritizing the test-time reduction for TIS is crucial. Moreover, the proposed analysis and related conclusions for the TIS also apply to the TRP due to reciprocity.

$$W\left(\theta_{i}\right)=\frac{c_{i}}{N}\left[1-\sum_{j=1}^{int(\frac{N}{2})}\frac{b_{j}}{4j^{2}-1}cos(2j\theta_{i})\right]$$

with

(5) $$b_{j}=\left\{\left.\begin{array}{c} 1,\\ 2, \end{array}\begin{array}{c} j=N/2\\ j<N/2 \end{array}\right\}\right.,c_{i}=\left\{\left.\begin{array}{c} 1,\\ 2, \end{array}\begin{array}{c} i=0\;\mathrm{or}\;N\\ otherwise \end{array}\right\}\right..$$

**Figure 2:**
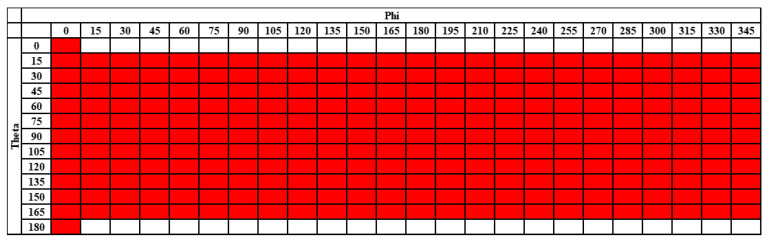
Illustration of the standard 15-degree TRP grid as reference.

The standard TRP measurement grid with 15-degree intervals in both theta and phi is illustrated in Figure 2.

The samples and weights for the Clenshaw–Curtis quadrature with this reference grid configuration (Δ*θ* = 15°) are illustrated in Table 1.

Currently, the fast TIS approaches being considered in the industry for test-time reduction include the following:

(1) TIS based on the device-reported RSS (received signal strength) values [14,15];
(2) Spiral scan method [14];
(3) The theta-dependent phi optimization [14];
(4) Device rotation during measurement [14];
(5) Single or multi-point offset in Anechoic Chambers (ACs) [14];
(6) Continuous-mode stirring in Reverberation Chambers [16,17,18];
(7) Grid points reduction.

Among all the aforementioned potential methods, the grid points reduction is the most effective and direct method, applicable to all bands and wireless technologies— that is, the original intention of this study, i.e., to determine optimal measurement grids with grid point reduction for OTA testing of 5G smart watches.

Apparently, from (1) and (2), the accuracy of OTA testing, specifically the calculation of TRP/TIS, depends heavily on measurement grid configurations. The more points and the more even the distribution, the higher the precision, and consequently, the longer the test time. To reduce test time, a compromise should be made between accuracy and the number of grid points required. Importantly, analyzing the influence of different grid configurations on OTA testing accuracy, as well as quantitatively evaluating relevant MU terms, are prerequisites. However, relevant work in this area has not been conducted or published yet and remains a major concern of the industry.

For the sake of simplicity, this study provides a quantitative influence analysis of measurement grid configurations on the accuracy of TRP calculations. The proposed approach and related conclusions presented here also apply to TIS testing. This impact is primarily reflected in deviations between TRPs obtained from different DUT orientations relative to the measurement grid. For some very coarse grids, these deviations can exceed 2 dB, which is unacceptable.

This impact originates from lower grid resolution, which lacks sufficient sampling precision for accurately reconstructing the DUT’s radiation patterns across various orientations. Consequently, for this impact and related MU analysis, the most realistic approach is to characterize the statistical distribution of TRP values across a large number of random DUT orientations relative to the measurement grid. Indeed, this analysis can be done in an experimental manner. However, it is required that the number of independent measurements be sufficient to achieve statistical significance. Considering the entire test time and resources required, including instruments, labor, chambers, etc., it is unrealistic for the industry to undertake such an experimental analysis on a large scale. Therefore, the relevant MU analysis proposed in this research is conducted through numerical simulations, wherein the relative orientation between the DUT (or, in other words, the reference patterns) and the measurement grid is altered randomly, and the standard deviation (STD) of TRPs for each measurement grid configuration is derived from a set of 10,000 random orientations. As a result, this STD is identified as the grid-dependent MU term, characterizing the impact of a given grid configuration on TRP calculation accuracy (or, OTA performance). The steps followed for the analyses are outlined below:

Step 1: import the reference pattern with very fine angular resolution, i.e., $\Delta \theta =\Delta \varphi =1^{\circ }$;

Step 2: determine the reference TRP based on the reference pattern with very fine angular resolution;

Step 3: apply 10,000 random rotations to the reference pattern, specifically, first rotating around the x axis, followed by the y axis and then the z axis in sequence;

Step 4: discretize the rotated reference pattern to the specific coarse grid, for example, with an angular resolution of 30°/45° in theta/phi;

Step 5: calculate and store the TRP value for the coarse grid being analyzed with each rotation;

Step 6: calculate the STD of the resulting 10,000 TRPs from the 10,000 random rotations to obtain the required MU.

## 3. Reference Patterns for MU Analysis of Different Grid Configurations

In this paper, a variety of coarse grid configurations are analyzed to compare their accuracy in reconstructing the reference patterns and subsequently to determine the related MUs. The reference patterns used in this research are passive patterns measured from a 5G commercial smart watch at different bands, with very fine angular resolution. The rationale for this operation is based on the following considerations:

(1) Based on the radiation behavior investigation conducted in CTIA [19,20], radiation properties among different smart watches from different manufacturers are highly similar, owing to their very limited space and working volume, especially the nearly identical usage scenario of wrist-worn devices. This has become a consensus in the industry. Therefore, in the CTIA Small Form Factor Device Inter-Lab Comparison Test Exercise, only one Vowor watch was used as the reference device. Different labs performed measurements using this reference device, and their testing results were compared. Furthermore, radiation behaviors of smartphones from different vendors are also considered highly similar by CTIA and 3GPP due to their highly similar usage scenario of handheld devices. Accordingly, only one or two smartphones were used as the reference devices in the CTIA and 3GPP Inter-Lab Comparison Test Exercise for smartphones [21,22,23];
(2) These patterns can represent the general radiation behaviors of typical 5G commercial smart watches, including the directivities, variations over angles, etc.

Accordingly, instead of analyzing the grid influence using individual radiation patterns from various devices and manufacturers case by case, the MU analysis for grid influence is performed using a single set of reference patterns. This ensures that the analysis and related conclusions are applicable to all cases while simultaneously reducing the workload and duration of the analysis.

**Figure 3:**
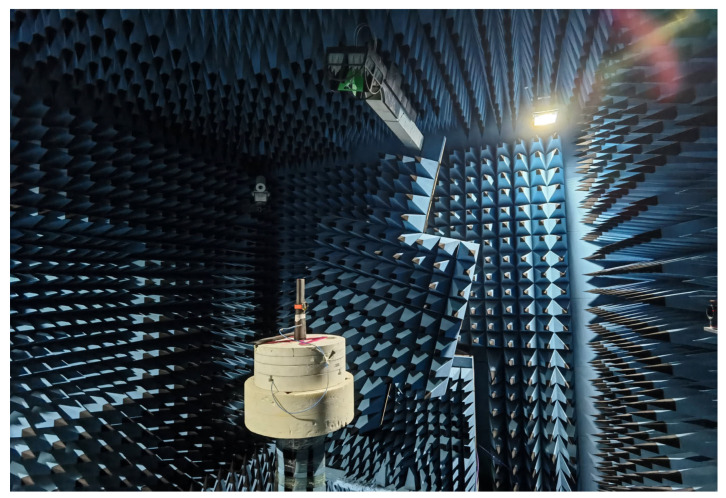
The measurement scenario of the reference patterns in the chamber.

**Figure 4:**
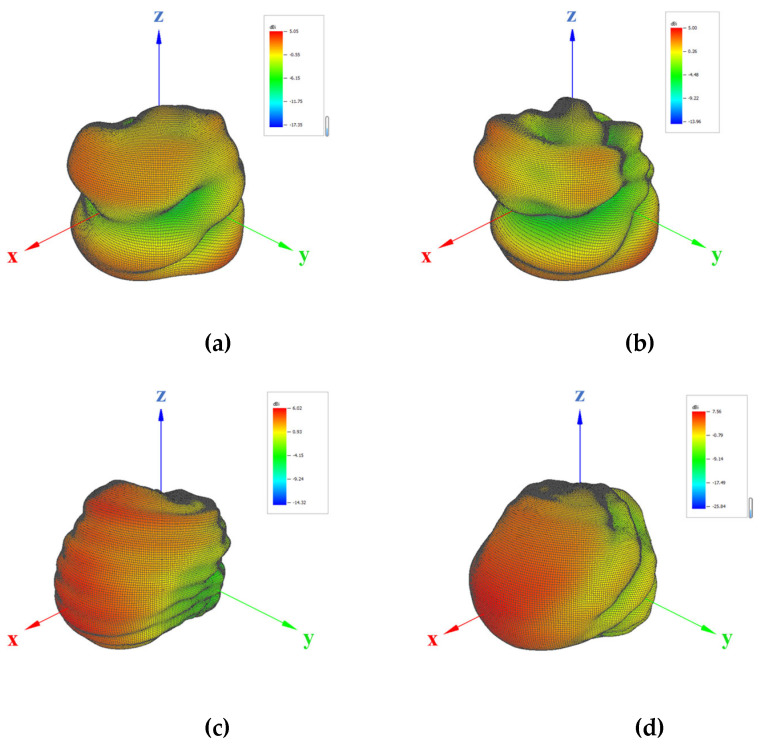

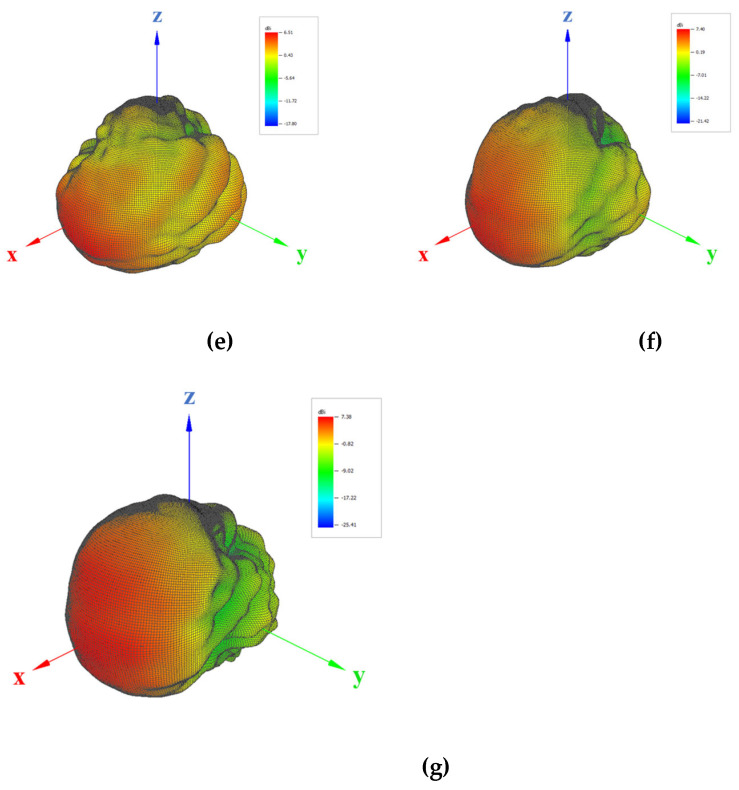
Illustration of reference patterns at different bands: (**a**) 836 MHz; (**b**) 900 MHz; (**c**) 1575 MHz; (**d**) 1950 MHz; (**e**) 2350 MHz; (**f**) 2450 MHz; (**g**) 2590 MHz.

The reference patterns are measured in wrist-worn condition, i.e., with the smart watch worn on the forearm phantom, as illustrated in Figure 3. During the measurement, both the E-field amplitude and phase values are measured and stored in the near field using the WPTC-L chamber from Rohde and Schwarz, while the reference patterns are obtained through a near-field to far-field transformation (NFTF). The adopted reference patterns for various bands under wrist-worn condition are illustrated in Figure 4, featuring a very fine angular resolution of ∆*θ* = ∆*ϕ* = 1*°*.

The measured directivities at different bands are listed in Table 2 for reference.

## 4. Simulated and Measured Results

**Figure 5:**
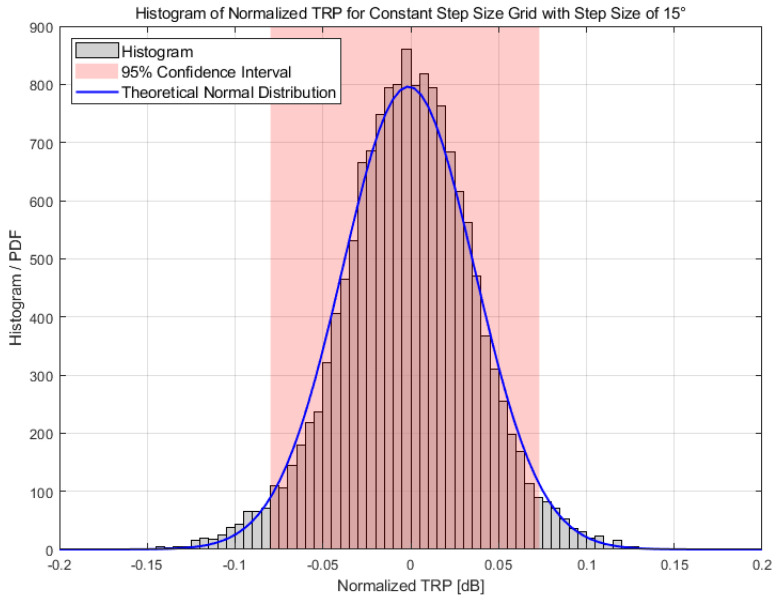
Sample histogram of the 10,000 normalized TRPs for the 15° constant step size measurement grid.

**Figure 6:**
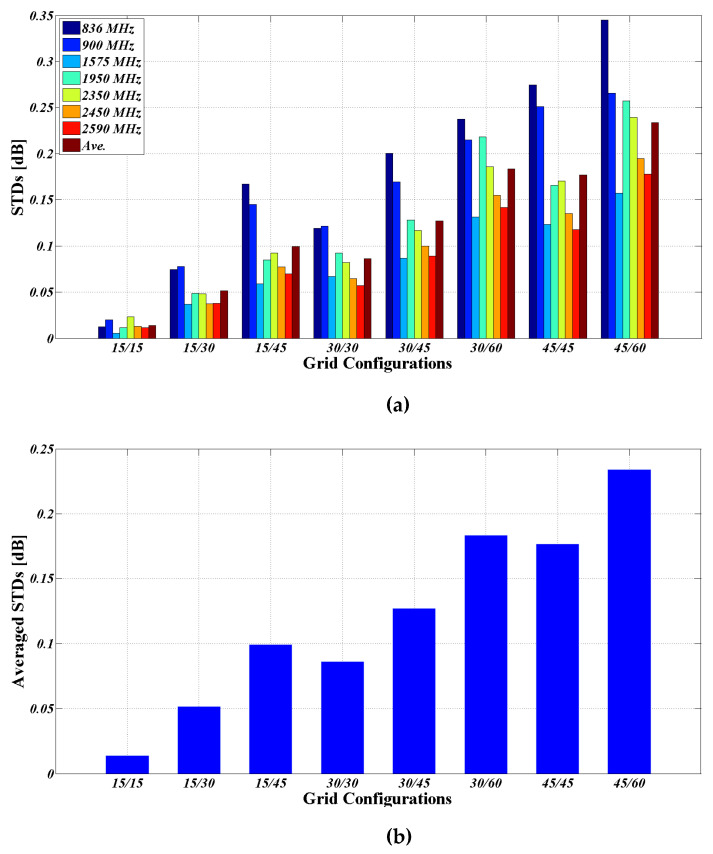
The comparison of calculated STDs: (**a**) at different bands; (**b**) highlighted illustration of the averaged STDs across all bands.

Based on the settings aforementioned, various constant step size grids with reduced angular resolution are analyzed. For the example of the 15° constant step size grid, the histogram for the normalized TRP is shown in Figure 5. The sample histogram of theoretical normal distribution is also provided for comparison. The histogram of the normalized TRP exhibits a normal distribution from the comparison. The calculated STDs at different bands are tabulated in Table 3 and also illustrated in Figure 6 for comparison.

The following observations could be obtained from the comparison:

Observation 1: with the same grid configuration, radiation patterns that exhibit larger variations over angles (i.e., the more lobed patterns) could have higher calculated STDs (i.e., higher MU for grid configuration);

Observation 2: typically, as the grid resolution decreases, the total number of grid points decreases, whereas the calculated STD increases. Furthermore, it is worth noting that the more evenly distributed grid configurations (where ∆*θ* and ∆*ϕ* are closer in value), even with fewer grid points, may exhibit smaller calculated STDs. For instance, the 15/45 grid with 90 grid points exhibits larger calculated STDs than the 30/30 grid with 62 grid points, across almost all bands;

Observation 3: at 836 and 900 MHz, the measured patterns exhibit significantly greater variations over angles, which can be attributed to the low radiation efficiency of lower band antennas in a very limited space. Moreover, these two bands are not the major bands used for smart watches;

Observation 4: for TIS testing, the 30/30 grid, which is the currently required TIS grid in both CTIA and 3GPP specifications, especially for smartphones and notebooks, corresponds to an average STD of 0.0862 dB across all bands being analyzed. From the test-time reduction perspective, the required minimum number of grid points can be reduced to speed up TIS testing, albeit at the expense of a proper relaxation of the realized MU limits. Accordingly, it is proposed that a proper relaxation in MU can be allowed for grid point reduction, such that the calculated STDs remain below 0.20 dB. In other words, it is recommended to adopt 0.20 dB as the resulting MU limit for TIS grids of 5G smart watches, which is approximately one-tenth of the currently defined expanded uncertainty limits for TIS testing. Compared to the benefit of approximately 60% grid points reduction, the expense of additional 0.12 dB in MU could be deemed acceptable and is thus recommended. Given that the expanded uncertainties for TRP/TIS testing are calculated using the root-sum-of-squares method, rather than being directly summed in dB, the impact of using the coarse grid on the final expanded uncertainty or the labs’ assessed MU could be significantly smaller than the increase in MU for the TIS grid itself, specifically, much smaller than 0.12 dB;

Observation 5: for TRP testing, the 15/15 grid—which is the currently required TRP grid in both CTIA and 3GPP specifications—corresponds to an average STD of 0.0138 dB across all bands being analyzed, implying that the impact of the currently defined grid on TRP testing accuracy can be considered negligible. Therefore, in pursuit of grid point reduction, it is recommended to adopt 0.10 dB as the maximum MU limit for 5G smart watch TRP grids. The allowance of additional 0.08 dB in the MU limit, specifically increasing from 0.0138 dB to 0.10 dB, could achieve a grid points reduction by approximately 75%. Similarly, an increase of 0.08 dB in the MU of the TRP grid could result in an increase in the final expanded uncertainty or the labs’ assessed MU of much smaller than 0.08 dB;

Observation 6: based on the recommended criteria above, the TIS and TRP grids could be reduced to 45/45 (26 points) and 30/30 (62 points), respectively, with around a 60% and 75% reduction in grid points, respectively. Given that the overall test time is proportional to the total number of grid points used, a significant reduction in test time can be achieved.

The TRP pattern data was collected for seven sample smart watches using standard settings and procedures from [14], covering over 50 bands supported by these devices. During the TRP testing, only mid-channels were included in this analysis, as the patterns for the low and high channels were deemed similar to those of the mid-channel. The TRPs obtained with the 15/15 grid and Clenshaw–Curtis quadrature are used as the baseline, which is assumed to be accurate. Consequently, the uncertainty associated with various coarse grids, expressed as the delta in TRPs, can be calculated accordingly.

The following Table 4 presents the comparison between measured and simulated STDs in the TRP for various coarse grid configurations. Good overall agreement could be observed, with the discrepancies falling within 0.06 dB. These discrepancies could be considered very small, given that during TRP testing, the UEs’ output power (i.e., EIRP) could vary by up to 0.5 dB (or even more) throughout the measurement process. As this happens, the radiation pattern will inevitably be changed. However, this variation in output power is not a measurement error, but rather a DUT-related variation that could be unavoidable. Indeed, this raises concerns about accuracy and could result in increased variation in measured TRPs (weighted sum of EIRPs), ultimately leading to a certain degree of discrepancy enlargement between the simulated and measured MUs. Nonetheless, this comparison effectively demonstrates the feasibility of the proposed MU analysis as well as related conclusions.

Furthermore, the use of these coarse grids will enable faster OTA testing, which will reduce the need for repositioning by over 50% or even eliminate the need for repositioning due to battery depletion or thermal limitations. When this occurs, the DUT should be removed from the chamber for cooling and charging. The corresponding reduction in repositioning MU will offset the additional MU incurred by using coarse measurement grids, resulting in a similar overall MU or potentially even an improved overall MU.

## 5. Conclusions

The objective of this research is to determine the optimal measurement grids for OTA testing of 5G smart watches, balancing accuracy with test time. The effect of various coarse grids on OTA performance, specifically the accuracy of TRP/TIS calculation, is analyzed quantitatively using reference patterns from a 5G commercial smart watch with very fine resolution across different bands. The related MU terms are then evaluated and determined quantitatively based on statistical analysis. Based on the STDs comparison, the reasonable MU limits for measurement grid configurations are recommended as the minimum number of grid points with STDs no more than 0.10 dB and 0.20 dB for TRP and TIS testing, respectively. Consequently, the TIS and TRP grids could be reduced from 30/30 (62 points) to 45/45 (26 points) and from 15/15 (266 points) to 30/30 (62 points), respectively, achieving over 60% and 75% reduction in grid points. Given that the overall test time is proportional to the total number of grid points utilized, significant reductions in test time can be achieved. Moreover, reducing the number of grid points could also reduce and even eliminate the repositioning-related MU, thereby realizing an overall MU similar to or potentially even improved over that of using the standard grids. The feasibility of the proposed MU analysis and the recommended grids is proven by experiments. Above all, the proposed analysis is applicable to measurement grid determination for any type of DUTs with specified radiation behavior.

## Figures and Tables

**Table 1:** Samples and weights for the Clenshaw–Curtis quadrature with Δ*θ* = 15°.

Clenshaw–Curtis Quadrature		
i	θ [deg]	Weights
0	0	0.007
1	15	0.0661
2	30	0.1315
3	45	0.1848
4	60	0.227
5	75	0.2527
6	90	0.262
7	105	0.2527
8	120	0.227
9	135	0.1848
10	150	0.1315
11	165	0.0661
12	180	0.007

**Table 2:** The measured directivities at different bands.

	836 MHz	900 MHz	1575 MHz	1950 MHz	2350 MHz	2450 MHz	2590 MHz
Directivities [dBi]	5.05	5.00	6.02	7.56	6.51	7.40	7.38

**Table 3:** Statistical analyses of 10,000 rotations for various constant step size grids [dB].

Angular Step Size		# of Unique Grid Points	836 MHz	900 MHz	1575 MHz	1950 MHz	2350 MHz	2450 MHz	2590 MHz	Ave.
Δ*θ*	Δ*φ*									
Δ*θ* = 15°	Δ*φ* = 15°	266	0.0125	0.0199	0.0052	0.0113	0.0234	0.0129	0.0117	0.0138
	Δ*φ* = 30°	134	0.0744	0.0779	0.0368	0.0487	0.0482	0.0372	0.0380	0.0516
	Δ*φ* = 45°	90	0.1668	0.1449	0.0590	0.0847	0.0924	0.0775	0.0700	0.0993
Δ*θ* = 30°	Δ*φ* = 30°	62	0.1191	0.1215	0.0669	0.0922	0.0820	0.0648	0.0570	0.0862
	Δ*φ* = 45°	42	0.2004	0.1694	0.0866	0.1280	0.1166	0.0999	0.0889	0.1271
	Δ*φ* = 60°	32	0.2373	0.2151	0.1315	0.2180	0.1859	0.1550	0.1417	0.1835
Δ*θ* = 45°	Δ*φ* = 45°	26	0.2748	0.2513	0.1233	0.1654	0.1703	0.1351	0.1179	0.1768
	Δ*φ* = 60°	20	0.3450	0.2656	0.1571	0.2574	0.2395	0.1946	0.1778	0.2339

**Table 4:** Comparison between measured and simulated STDs [dB].

Deltas	Sin Theta		Clenshaw–Curtis				
	15/15	30/30	15/15	15/30	30/30	30/60	45/45
Max	0.1	0.27	0	0.06	0.26	0.36	0.82
Min	−0.02	−0.4	0	−0.03	−0.5	−0.6	−0.36
Delta (max–min)	0.12	0.67	0	0.09	0.76	0.96	1.18
Meas. STDs	0.02	0.11	0	0.06	0.11	0.24	0.21
Simulated STDs	-	-	0.0138	0.0516	0.0862	0.1835	0.1768

## Data Availability

Dataset available on request from the authors.
